# Psychological Impact on the Nursing Professionals of the Rioja Health Service (Spain) Due to the SARS-CoV-2 Virus

**DOI:** 10.3390/ijerph18020580

**Published:** 2021-01-12

**Authors:** Pablo Del Pozo-Herce, Rebeca Garrido-García, Iván Santolalla-Arnedo, Vicente Gea-Caballero, Pablo García-Molina, Regina Ruiz de Viñaspre-Hernández, Francisco José Rodríguez-Velasco, Raúl Juárez-Vela

**Affiliations:** 1 Department of Psychiatry, Fundación Jiménez Díaz University Hospital, 28040 Madrid, Spain; pablo.pozo@quironsalud.es; 2 Group of Research in Sustainability of the Health System, Biomedical Research Center of La Rioja (CIBIR), 26006 Logroño, Spain; rgarridog@riojasalud.es (R.G.-G.); reruizde@unirioja.es (R.R.d.V.-H.); raul.juarez@unirioja.es (R.J.-V.); 3 Rioja Health Service, Najera Health Center, 26300 La Rioja, Spain; 4 Department of Nursing, GRUPAC, University of La Rioja, 26006 Logroño, Spain; 5 Nursing School La Fe, Adscript Center Universidad de Valencia, 46010 Valencia, Spain; 6 Research Group GREIACC, Health Research Institute La Fe, 46026 Valencia, Spain; 7 Department of Nursing, University of Valencia, 46010 Valencia, Spain; pablo.garcia-molina@uv.es; 8 Grupo Asociado de Investigación en Cuidados, Fundación Incliva, 46010 Valencia, Spain; 9 Department of Nursing, Faculty of Medicine, Badajoz University of Extremadura, 06006 Extremadura, Spain; fcorodriguezv@unex.es

**Keywords:** nursing, mental health, COVID-19, SARS-CoV-2, coronavirus

## Abstract

Background: The COVID-19 pandemic is a public health emergency that has affected health professionals around the world, causing physical and mental exhaustion with a greater probability of developing mental disorders in professionals who provide healthcare. Objective: The objective of this study was to know the psychological impact of the SARS-CoV-2 virus on the nursing professionals working for the Rioja Health Service. Methods: We conducted an observational and descriptive cross-sectional study. The nursing staff at the Rioja Health Service were invited to respond to a self-administered questionnaire between June and August 2020. Results: A total of 605 health professionals participated in the questionnaire; 91.9% were women, 63.14% were registered nurses, and 36.28% were auxiliary nurses. Risk factors for mental health professionals were identified in more than 90% of nurses (*p* = 0.009), affecting their psychological state with feelings of exhaustion, emotional overload (*p* = 0.002), and less use of coping strategies among women. Younger professionals with less experience had higher levels of stress compared to those with more than five years of experience, who showed a progressive reduction in the impact of stressors (*p* < 0.001). Professionals with dependent family members presented higher levels of emotional overload and coping problems (*p* = 0.009). Conclusion: The COVID-19 pandemic has had a significant psychological impact on health professionals in terms of stress, emotional well-being, and the use of coping strategies. Female health professionals with dependents, a temporary contract, and less work experience have been more psychologically affected.

## 1. Introduction

In December 2019, the first outbreak of the SARS-CoV-2 virus, known as COVID-19, was reported in Wuhan, China, with rapid expansion throughout the world [1]. Soon, it was declared an international public health emergency and recognized by the World Health Organization (WHO) as a global pandemic on 11 March, 2020. Across the world, the virus has had an intense negative impact, the rapid increase in demand for health facilities and professionals has caused many healthcare systems to not function effectively [2]. Since the start of the pandemic to 18 November 2020, 1,291,808 cases of COVID-19 have been reported in Spain [3]. Healthcare workers have suffered enormous pressure with long work shifts, [4] inadequate conditions, high risk of infection, ethical dilemmas when allocating scarce resources to equally needy patients [5], lack of specific skills, frustration [6], social stigmatization, isolation, concern about spreading the virus to their families [4,7], lack of contact, and lack of social support [8]. These factors have forced many health professionals to make sensible changes in their daily lives that compromise their health and psychological well-being, as a consequence of physical and mental exhaustion [2,9,10,11].

These factors can give rise to different levels of psychological pressure that can trigger health problems such as feelings of loneliness, helplessness, stress [12], anguish [9], anxiety [6,13], depressive symptoms [4], insomnia, denial, anger, fear, irritability, sleep disorders [2], burnout syndrome [5], and even risk of suicide [10]. This pressure exceeds the psychological and emotional limits of health professionals, increasing the risk of psychological suffering with a higher probability of developing the abovementioned mental disorders [6,11,12], in addition to vicarious traumatization related to compassion towards the patients cared for [14]. Further, post-traumatic stress [5,15,16] can be a long-term consequence of this pressure [7].

According to studies in the first months of the pandemic, between 71% and 89% of health workers that were in high-risk situations, had suffered psychological symptoms [2,5,6]. About half reported depressive symptoms (50.4%) and anxiety (44.6%), respectively, whereas 34% reported insomnia [5,8,9,10]. In total, 34.4% of nurses and physicians claimed to have mild disorders, 22.4% had moderate disorders, and 6.2% had severe disorders [5]. This especially affected young women, which is consistent with previous pandemics [4,17,18]. Direct and continuous contact with infected patients influences anxiety; these professionals experienced higher anxiety scores than those who had not met directly with positive patients [19]. Higher incidence values were found in female nurses compared with male physicians because they tend to have longer and closer contact with patients [12]. To find a solution, 36.3% of professionals searched for and accessed psychological materials, such as books on mental health, whereas 50.4% sought psychological resources available through the media, such as online information on mental health and coping methods. Furthermore, 17.5% of the sampled healthcare workers participated in professional counseling or psychotherapy [5]. About 20% of health professionals suffered stigmatization or discrimination from some people with whom they interacted at work, and up to 49% perceived similar feelings when interacting with the population in their daily life outside of work [4,5].

It should be noted that managers of health systems need to maintain the physical, mental, and professional integrity of their workers, especially nurses, who have witnessed a high number of deaths, illnesses, and disabilities caused by COVID-19 [2]. This should involve global strategies to protect the health of these workers by maintaining mental health and especially controlling depressive symptoms, anxiety, and suicidal ideations, as well as early identification of secondary psychosocial factors that can generate stress [12]. Most studies have indicated that the psychological safety of health personnel is an essential condition to providing quality care to patients [7], and it should be a chief concern to develop strategies to prepare, educate, and strengthen the mental health of the affected population [2,5].

To protect professionals, it is necessary to obtain information about the reality of the psychological impact caused by this pandemic, and to relate this new knowledge with that generated in other previous similar pandemics (such as the SARS-CoV-1 and MERS pandemics). In this way, optimal intervention strategies can be designed to moderate or reduce the risks and consequences of mental health deterioration in nurses. There is a need for more resilient professionals who take advantage of their personal resources to face health crises with greater personal strength. For this reason, the objective of this study was to uncover the psychological impact of the SARS-CoV-2 virus on nursing professionals at the Rioja Health Service (SERIS).

## 2. Materials and Methods

### 2.1. Study Design

We performed an observational and descriptive cross-sectional study carried out between June and November 2020.

### 2.2. Population and Scope of the Study

The study was carried out in the autonomous community of La Rioja (Spain). According to data from the government of La Rioja, for a sample with a 95% confidence interval and an alpha error of 5%, the minimum sample calculated was 310 subjects. The questionnaire was sent to all SERIS workers (highlighting registered nurses and nurse auxiliary) using an internal mailing service. We analyzed the results received during the time the questionnaire was available between 19 June and 6 August 2020, as long as the minimum sample size was exceeded.

### 2.3. Study Variables

The main variables in the study population were stressors, perceived emotions, and coping strategies. The secondary variables included the demographic data of the professionals (age by intervals, sex, marital status, number of children, dependents) as well as data related to the job, among others (professional category, type of contractual relationship with the company (e.g., permanent, temporary) and years of professional experience, see Table 1.)

### 2.4. Data Collection Instrument and Procedure

For data collection, a self-administered questionnaire was prepared that collected information on the response of the health system and its professionals in the COVID-19 crisis.

The objective of the study was defined and characterized by a bibliographic review to know what had been published so far, as a starting point to carry out the process of questionnaire construction. With the information obtained, two of the researchers analyzed and defined three dimensions of study that contributed to a better selection and operationalization of the variables.

Once the first version of the questionnaire was made, it was sent to two independent experts for questionnaire validation. The reviewers evaluated the following aspects: structure of the general design of the questionnaire; the number of questions; structure and content of each question, and their interpretation; problems with the application of the questionnaire; understanding the questions and operations of the instrument that concerned the language or writing of the items; and the ease of interpretation for each item. With their contributions, the final questionnaire of 47 items was prepared. The questionnaire was prepared in a digital version using the Microsoft Forms^®^ tool for digital dissemination. To increase methodological control and avoid duplication, all similar questionnaires carried out on the same date and within one minute were filtered and eliminated. IP filtering was not used, as it prevented access to independent responses if users were connected to the Internet from the services of major institutions.

In the survey form, a combination of forced-choice (i.e., Never, Sometimes, Almost Always, Always) and comments were used. The forced-choice questions were mandatory.

The validation of the internal consistency of the questionnaire was determined through the calculation of Cronbach’s alpha coefficient, which allowed us to check the internal context of each item. The test was considered acceptable when the alpha value was equal to or greater than 0.7.

The questionnaire itself had 3 important parts: the stressors dimension (which collected items from 14 to 34), the perceived emotional dimension (which collected items 35 to 38), and the coping dimension (which collected items 39 to 42). The reliability of this structure was acceptable where Cronbach’s alpha is shown for each of the original dimensions, see Table 2.

To observe the differences in different stressors, it was decided to quantify the instrument. To do this, a total score was created for each dimension that took values from 0 to 3 or 0 to 5, depending on the item to be assessed (on the Likert scale). First, 0 was the most positive answer and 3 or 5 was the most negative. Two questions were categorized in another way: the first was related to whether one had been infected by the SARS-CoV-2 virus. If a participant answered with 0, then they indicated that they had not been infected whereas 1 denoted that they had been infected. The second question corresponded to item 43. If a participant answered with 0, then they had not requested psychological help on the indicated telephones; if they answered 1 then it indicated that they had not, but would like to, and; 2 indicated that they had requested help.

Thus, the first dimension (stressors) adopted values between 0 and 81 (questions 20, 28, 29, and 30 were not included in the total score since only a small group of the initial sample answered).

The second dimension (perceived emotions) adopted values between 0 and 20, since the Likert scale in the four questions made up this dimension of values from 0 to 4.

The third dimension (coping strategies) adopted values between 0 and 16, since the Likert scale in the four questions made up this dimension of values from 0 to 3.

The questionnaire was available throughout the validity period of the survey (between 19 June and 6 August 2020). This period coincided with the mitigation phase of the crisis, a time that seemed ideal not to overload professionals even more in the acute phase of the pandemic, but having very recent experience, and therefore it was more faithfully remembered.

### 2.5. Statistical Procedures and Analysis

The description of the quantitative values was made using descriptive statistics (mean and standard deviation). Since the data distributions were not Gaussian, other robust statistics such as the median and interquartile range, as well as the maximum and minimum values, were also indicated. The distributions of categorical variables were described using absolute and relative frequencies of the distribution.

For the inferential analysis of the data, the items of the questionnaires between the groups formed by demographic variables were described by means and standard deviation. The t-Student or Mann–Whitney U test was used to estimate the relationships between variables (in the case of comparing two groups depending on whether the variable has a normal distribution or not), or utilizing the one-way ANOVA test or Kruskal–Wallis H test (in the case of comparing more than two groups).

To assess the validity and structure of the three dimensions once its Cronbach has been analyzed and indicated good internal consistency, an exploratory factor analysis (EFA) was performed (Table 3) with the 3 dimensions, all the components with values >1 are extracted (Kaiser’s rule) and they also explained 84.48% of the variance. The values of the Kaiser-Meyer-Olkin test (KMO) reflected a good factorial model. Berlet’s sphericity in addition, implied a good factorial model. Confirmatory Factor Analysis (Table 4) was carried out in order to analyze Akaike’s Information Criterion (AIC), Bayesian Information Criterion (BIC), Comparative Fit Index (CFI), Tucker-Lewis Index (TLI), Standardized Root Mean Squared Residual (SRMR), and the Coefficient of Determination (CD).

### 2.6. Ethical Considerations

The survey was anonymous and did not collect personal data or devices that could identify the informant. There was no trace (Internet protocol (IP) or any other) of the respondents. The information was treated confidentially and anonymously since they had dissociated data, following the Data Protection Regulation (EU) 2016/679 of the European Parliament and the Spanish Organic Law 3/2018. [20]. The researchers did not declare any type of ethical, moral, or legal conflict, nor did they claim to have received financial compensation of any kind. The participants did not receive any type of compensation for answering the questionnaire, as it was voluntary.

## 3. Results

### 3.1. Descriptive Results

In total, 605 participants answered the questionnaire (response rate = 37.88%). Interestingly, 91.9% of the study participants were women. The mean age was mostly between 36 and 55 years (56.5%). Most of the participants were married (56.2%). Some participants (18.68%) had dependents in their charge. Further, 63.80% were RN, whereas 36.2% were AN. Concerning the type of service provision, 52.23% were civil servants. Almost 60% had more than 15 years of professional experience (Table 1).

### 3.2. Descriptive Data

**Figure 1:**
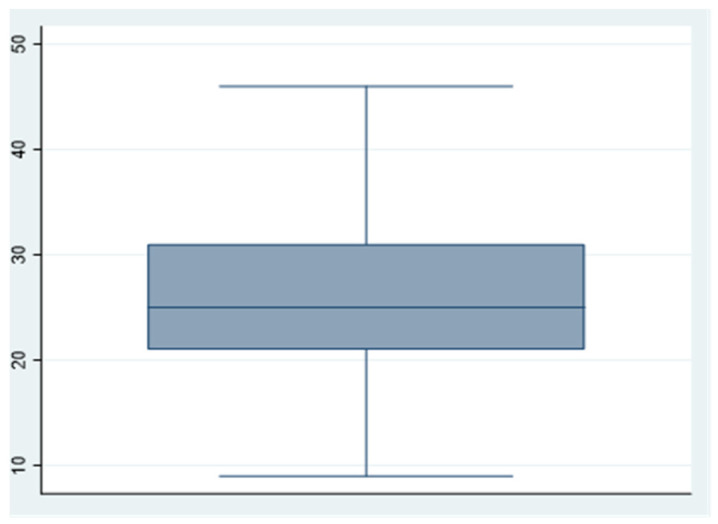
Stressors.

**Figure 2:**
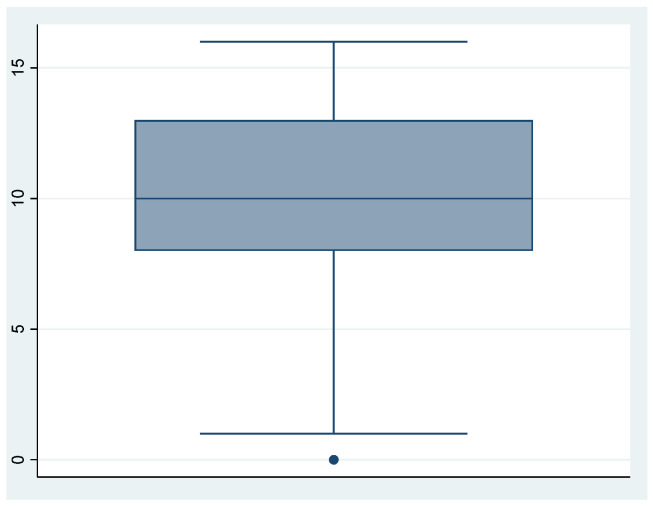
Perceived emotions.

**Figure 3:**
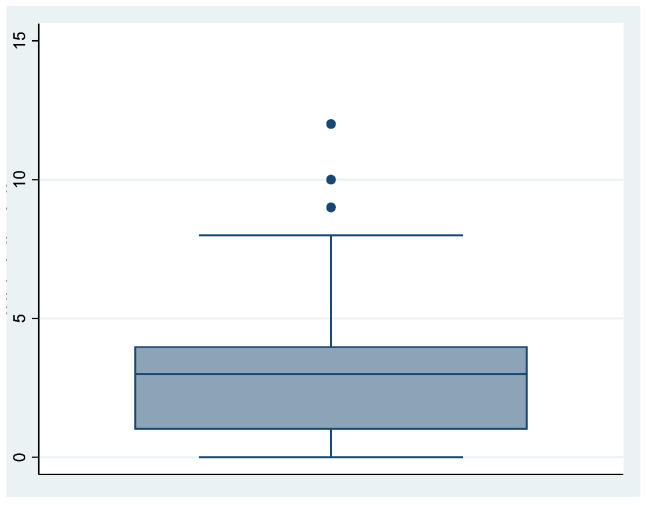
Coping strategies.

We can see the obtained scores from the questionnaire in Table 5 and Figure 1, Figure 2 and Figure 3.

### 3.3. Comparative Analysis of the Variables

The analysis of the sex variable in relation to the study dimensions (i.e., stressors, perceived emotions, and coping strategies) shows a mean of the stressors in women of 25.96 ± 6.75 and 23.31±7.47 in men. A value of *p* = 0.009 was estimated, and these differences were statistically significant. We observed significance (*p* = 0.002) with respect to the mean of emotions perceived in women (10.34 ± 3.68) and men (8.67 ± 4.08). Regarding the analysis of the dimensions and the variable “dependents”, an average of perceived emotions with dependents (11.03 ± 3.68) and without dependents (10.02 ± 3.72) was observed, with a *p*-value = 0.009. These differences were statistically significant. The analysis of the employment relationship with the company and assessing the relationship of being or not being a permanent staff member with the dimensions under study showed an average amount of stress factors in permanent staff (24.54 ± 6.53) compared to an average amount (27.14 ± 6.87) in temporary personnel. As such, *p* < 0.001 was estimated, and these differences were considered statistically significant. Significance was also observed in the perceived emotional dimension. It was 14.02 ± 3.77 in fixed staff versus 15.10 ± 3.39 in temporary staff. For the coping dimension, a mean of 2.51 ± 1. 53 was observed in permanent staff and 3.27 ± 2.20 in temporary staff, with an estimated significance of *p* = 0.035 for the emotional dimension and *p* = 0.005 for coping strategies. Regarding the work experience variable, the mean of the stressors in professionals with an experience of 1 to 5 years was 28.46 ± 7.06. From 6 to 10 years, the mean was 27.57 ± 6.17, from 11 to 15 years the mean was 26.15 ± 6.62, and over 15 years the mean was 24.98 ± 6.75. A *p*-value of 0.001 was estimated, and these differences were considered statistically significant. The analysis of the age of the sample reflected statistical significance for the impact of stress factors. A p-value of 0.001 was estimated, with a mean for the stress factors in people under 25 years to be 27 ± 5.77 For those aged 26 to 35 years, it was 28.24 ± 6.45, and for those aged 36 to 45 years it was 26.95 ± 6.67. Further, for those aged 46 to 55 years, it was 24.71 ± 6.97 and 23.27 ± 6.45 for those aged over 55 years.

Table 6 shows the results of the comparative analysis between the independent variables (sex, dependents in charge, type of employment relationship, time of experience, and age) and the dependent variables (stressors, perceived emotions, and coping).

The sex variable showed statistically significant differences concerning stressors and perceived emotions. The variable that corresponded to people with dependents was statistically significant for perceived emotions. The type of employment relationship presented statistically significant differences between the three dimensions. The time of experience showed statistically significant differences in all age groups to stressors. Age presented statistically significant differences in the dimension of stressors.

## 4. Discussion

The study of psychological impacts experienced by nursing professionals during a pandemic has a limited history, especially in Spain, and scientific publications at the national and international level are still scarce. Faced with this reality, the present study aimed to analyze the impact of the pandemic on nursing professionals (RN and AN), assessing the significance of variables typically studied, i.e., age, sex, family situation, type of employment contract, experience professional, analyzing professional exposure to stressors, emotional response, and coping capacity.

The designed questionnaire obtained a reliability coefficient according to Cronbach’s alpha that varied from 0.79 to 0.88 depending on the dimension under study. The internal consistency within each of the dimensions was classified as “very good”, which allowed us to trust the results of the study and we highlighted their robustness [21,22,23].

The results reflect that psychological risk factors were present in a high percentage of professionals under study. Factors were related to the fear of becoming infected or infecting loved ones. Moreover, the fear of making mistakes, as well as not giving adequate physical and/or psycho-emotional care to a patient’s needs, were factors present in practically the entire sample. More than 90% of the nursing professionals reported that the development of their work activity during the pandemic impacted their psychological state, with feelings of physical exhaustion and emotional overload. Previous studies at an international level correlated to this type of pandemic with high levels of psychological symptoms [24], among which the suffering of anxiety, stress, depression, and sleep disorders stands out [25,26].

In relation to emotional overload, the sample scores showed feelings of sadness, rumination, negativism, and emotional destabilization. These feelings were consistent with those described by other authors in different samples of professionals during the COVID19 pandemic [2,4]. At the level of coping strategies, less than 5% of professionals requested psychological support, but the sample scores were high in coping with problems and self-critical analysis. Professionals aware of the psychological impact, sought help in prepared materials, bibliographies, and psychological resources available online. In a very small percentage, they requested specialized external help [5].

Regarding the analysis of the organizational components at the level of human, material, and coordination resources, the perception of the sample in a high percentage, according to the regional study service (SERIS), refers to the development during the pandemic of coordinated work, with the availability of appropriate material resources and staff according to needs. These elements can be understood as protective factors against the psychological risks of professionals. The concern of health system managers to maintain the psychophysical integrity of health professionals during the pandemic is reflected as a result of different studies [2]. The psychological safety of health personnel is perceived as an essential condition to provide quality patient care [5,7,12]. In the analysis of the psychological impact, the study reflects differences by sex, a greater presence of stressors, greater emotional burden, and less use of coping strategies among the women in the sample, results that are in line with other previous studies at an international level [13,25]. The role of the caregiver within the family home, which has been developed by women in our society throughout history, can be related to these results. The emotional burden and the perceived risk factors can be associated with the perception of COVID-19 fear and its impact on the family. Fear is an adaptive and natural human response to threatening situations, and SARS-CoV-2 is currently experienced as a threat, generating this feeling, in many cases, due to misinformation and a lack of knowledge based on evidence. It associates fear with feelings of vulnerability, loss of control, and concern for one’s own health, as well as the health of the family environment [27,28]. In this sense, the professionals in the sample with dependents reveal, along the same lines, higher levels of emotional charge, stressors, and coping problems. A study carried out in our country showed that living with people with chronic diseases increased the psychological impact on healthcare professionals due to the fear of contagion [27].

Regarding the repercussion of the impact and the age of the sample, older nursing professionals showed less affectation for the first stressors under study. As the age of the sample increased, the impact of these stressors decreased. This difference was usually statistically significant (*p* < 0.001). Other studies support these results [17], relating them to less work experience, greater vulnerability, and less adaptation to an overloaded and fickle healthcare environment. The analysis of the experience and labor relationship (permanent/temporary) reveals an experience of more than five years on the job showed a progressive reduction of stress. These differences consider work experience to be statistically significant (*p* < 0.001). Likewise, a contract with greater consolidation in the administration significantly reflects less stressors (*p* < 0.001), less emotional overload (*p* = 0.035), and a greater coping capacity (*p* = 0.005). Different studies have linked older age, experience, and consolidation in employment with the development of coping strategies that reduce stress, emotional overload, and the ensuing impacts of such stressors [2,7,17].

Similar epidemics, such as SARS-CoV-1 and MERS-CoV permitted us to understand similarities in pandemic-related psychological damage. In the SARS-CoV-1 epidemic, health professionals were affected by the fear of contagions and experienced high levels of depression, anxiety, fear, distress, and post-traumatic stress [29]. On the other hand, health professionals who were on the front lines of the MERS epidemic experienced greater symptoms of post-traumatic stress disorder [30].

It is surprising to note how similar experiences and complications during other pandemics did not prevent psychological consequences in this pandemic for healthcare professionals. As such, it is possible to determine the main strengths of this study, i.e., its timeliness, its results pertaining to the psychological impact of COVID-19, practical implications, potential application of intervention protocols that could reduce the psychological impact on nursing teams (especially for women, younger professionals, and less work experience). In line with the conclusions of other studies, we found it necessary to intervene in order to improve information and training regarding COVID-19, enhance security measures, guarantee professionals’ functional health stability with regard to sleep/rest, nutrition/metabolism, stress tolerance, role/relationships, protecting the family, and offering professional psychological support from occupational risk prevention services. Intervening early in the prevention of psychological risks that affect these professionals is essential to provide quality care to patients, maintain the system’s sustainability, and generate greater resilience for professionals in the face of stressful situations that may affect patients in the future [2,5,7,27].

## 5. Conclusions

Our study has shown that the COVID-19 pandemic has had a significant psychological impact on health professionals, both in terms of stress, emotional well-being, and the use of coping strategies. In our context, female health professionals with dependents, a temporary contract, and less work experience were more psychologically affected than others. Future research can be based on these results to design strategies to prevent the psychological impact of pandemics, or high-stress situations in healthcare systems.

## 6. Limitations

Since this is a descriptive study, we did not use a statistical projection to infer associations. Regarding sex, the idiosyncrasies of nursing professionals in our country should lead us to estimate the results of differences by sex with some reserve, due to the distribution of the sample with 91.9% of women versus 8.1% of men. Although an attempt has been made to avoid duplication through the specified methodology, it has not been possible to establish IP filtering due to the loss of subjects carrying out the questionnaires through the same network in the health institutions. Parallel control procedures through cookies could be implemented in future studies

## Figures and Tables

**Table 1:** Sociodemographic characteristics.

Characteristics	
Age	*n* (%)
<25 years old	38 (6.28)
26–35 years old	102 (16.86)
36–45 years old	159 (26.28)
46–55 years old	183 (30.25)
>55 years old	123 (20.33)
Sex	
Men	49 (8.1)
Women	556 (91.9)
Marital status	
Single	179 (29.58)
Married	340 (56.2)
Divorced	59 (9.75)
Widower	5 (0.83)
Other	22 (3.64)
Children	
None	220 (36.36)
One	121 (20)
Two	223 (36.86)
Three or more	41 (6.78)
Dependents in my charge	
Yes	113 (18.68)
No	492 (81.32)
Category	
Registered Nurse	386 (63.80)
Nurse Auxiliary	219 (36.20)
Type of employment relationship	
Civil servant	316 (52.23)
Not civil servant	289 (47.77)
Laboral Experience	
<1 year	33 (5.46)
1–5 years	82 (13.55)
6–10 years	61 (10.1)
11–15 years	67 (11.07)
>15 years	362 (59.83)

**Table 2:** Response and Score.

Item	a. Never	b. Sometimes	c. Almost Always	d. Always
**Dimension 1. Stressors** **(*N* = 605 Cronbach’s alpha = 0.8828)**				
14. I felt that I had material in the unit that guaranteed my safety in the care of patients infected or possibly infected by COVID-19.	10	212	264	119
15. Did I feel a lack or shortage of knowledge related to COVID-19 infection when providing care to patients?	38	368	153	46
16. I was afraid of making mistakes and failures in the care of patients infected with COVID-19.	31	332	147	95
17. I felt helpless when I saw a COVID-19 patient who was not progressing satisfactorily.	31	156	178	240
18. I felt that I did not pay attention to the psycho-emotional needs of COVID-19 infected patients.	105	300	138	62
19. I thought that the care I was providing was not sufficiently humanized.	116	281	141	67
20. I have had difficulty with a service with new techniques and procedures that I did not previously control and it affected my personal stability. Check only for people who have been relocated or hired because of the health crisis	122	240	60	27
21. Coordination and teamwork were observed during the work shifts.	13	191	284	117
22. There was enough staff in the unit where I worked during the pandemic.	87	150	207	161
23. I felt that the rest time I had was sufficient during the work shift.	105	266	134	100
24. I felt that I had an orderly rotation.	73	122	138	272
25. I have thought about the possibility of getting infected by COVID-19 during my shift.	11	233	159	202
26. I have thought about the possibility of being a probable asymptomatic carrier.	25	288	126	166
27. I tested positive for COVID-19.	Yes: *n* = 95		No: *n* = 510	
28. What level of stress was generated by being positive in COVID-19. Answer only people who have been positive. Score 1 to 5	3.38			
29. In what way has the disease passed? Answer only if you have been given positive in the test.				
a. Asymptomatic.	*n* = 34			
b. Mild symptoms without requiring hospital admission.	*n* = 58			
c. Serious symptoms that have required hospital admission	*n* = 3			
30. I have been afraid of infecting the people I live with. Answer only if you live with more people.	7	81	95	278
31. The situation I found myself in during the pandemic at work has affected my psychological state.	38	319	124	124
32. At the end of the shift I felt physically exhausted	5	154	232	214
33. At the end of the shift I felt emotionally overloaded.	10	146	197	252
34. I managed to fall asleep easily and my rest was restorative.	126	332	115	32
**Dimension 2. Perceived Emotions** **(*N* = 605 Cronbach’s alpha = 0.8379)**				
35. I often felt that I did not spend enough time on my feelings and emotions. Score 1 to 5	3.73			
36. I have felt sad and have thought about things too much. Score 1 to 5	3.91			
37. I did not feel able to think about the positive aspects of things. Score 1 to 5	3.35			
38. I believe that this crisis has changed me and destabilized me on an emotional level. Score 1 to 5	3.22			
**Dimension 3. Coping Strategies** **(*N* = 605 Cronbach’s alpha = 0.7917)**				
39. I have tried to avoid facing certain problems.	189	327	69	20
40. I have avoided expressing my emotions with my co-workers.	183	317	83	22
41. I have answered or spoken badly to my peers to release my pent-up emotions.	332	254	15	4
42. I preferred not to analyze the situation when I was doing something wrong, without performing self-criticism and continuing with my tasks.	365	212	25	3

**Table 3:** **Exploratory Factor Analysis** (EFA) Analysis. Varimax Rotation. (First part) Variance and Kaiser-Meyer-Olkin-Test (KMO) (Second part).

Varimax Rotation	Dimension 1	Dimension 2	Dimension 3
p14	0.4962		
p15	0.5111		
p16	0.5338		
p17	0.3039		
p18	0.4732		
p19	0.4510		
p21	0.4412		
p22	0.6506		
p23	0.5520		
p24	0.4990		
p25	0.6030		
p26	0.6107		
p27	0.2418		
p31	0.2444		
p32	0.2434		
p33	0.2454		
p34	0.3034		
p35		0.6236	
p36		0.8015	
p37		0.7273	
p38		0.7342	
p39			0.4451
p40			0.3147
p41			0.3404
p42			0.4451
**Kaiser-Meyer-Olkin Test (KMO)**			
**Item**			**Value**
p14			0.7715
p15			0.8097
p16			0.8829
p17			0.9073
p18			0.7837
p19			0.8004
p21			0.7471
p22			0.8049
p23			0.8221
p24			0.8283
p25			0.8403
p26			0.7798
p27			0.4910
p31			0.9431
p32			0.8555
p33			0.8639
p34			0.9423
p35			0.9244
p36			0.8950
p37			0.9163
p38			0.9220
p39			0.8823
p40			0.8640
p41			0.8683
p42			0.7160

Dimension 1, stressors items from 14–34. Dimension 2, perceived emotions items from 35–38. Dimension 3, coping strategies items from 39–42. Items from 1–13 were related to sociodemographic questions and were not analyzed in EFA and CFA.

**Table 4:** Confirmatory Factor Analysis (CFA) Analysis.

Analysis Value		
RMSEA	Root Mean Squared Error of Approximation	0.098
AIC	Akaike’s Information Criterion	40,223.5
BIC	Bayesian Information Criterion	40,611.2
CFI	Comparative Fit Index	0.969
TLI	Tucker-Lewis Index	0.935
SRMR	Standardized Root Mean Squared Residual	0.059
CD	Coefficient of Determination	0.994

**Table 5:** Psychometric characteristics of the dimensions.

Variable	N	Mean	SD	Min.	Max.
Dimension 1: Stressors	605	25.7438	6.8417	9	46
Dimension 2: Perceived emotions	605	10.20826	3.7340	0	16
Dimension 3: Coping strategies	605	2.712397	1.7717	0	12

**Table 6:** Comparative Analysis.

	Stressors			Perceived Emotions			Coping Strategies.		
	C	95% IC	*p*-Value	C	95% IC	*p*-Value	C	95%IC	*p*-Value
Sex									
Woman	3.02	1.08–4.96	0.002	1.57	0.49–2.65	0.004			
Men									
Dependents in my charge									
Not									
Yes	1.19	(−0.20)–2.59	0.094	0.94	0.17–1.72	0.017			
Type of employment relationship									
Civil servant	−2.09	(−3.36)–(−0.81)	0.001	−0.45	(−1.44)–0.54	0.374	−0.18	(−0.66)–0.29	0.455
Not civil servant	0.17	(−1.83)–2.17	0.868	0.95	0.16–1.74	0.018	0.45	(−0.03)–0.9	0.069
Laboral experience									
<1 year									
1–5 years	5.79	3.13–8.46	0.000	2.19	0.71–3.68	0.004	0.26	(−0.50)–1.02	0.506
6–10 years	4.99	2.18–7.79	0.001	2.18	0.62–3.73	0.006	0.69	0.32–1.07	0.000
11–15 years	4.78	1.90–7.66	0.001	1.60	0.06–3.15	0.042	0.17	(−0.73)–1.06	0.716
>15 years	4.19	1.55–6.83	0.002	1.75	0.43–3.08	0.009	0.15	(−0.70)–1.00	0.727

## References

[1] World Health Organization Coronavirus Disease 2019 (COVID-19) Situation Report. https://www.who.int/docs/default-source/coronaviruse/situation-reports/20200121-sitrep-1-2019-ncov.pdf?sfvrsn=20a99c10_.

[2] Moreira W.C., de Sousa A.R., de Sousa Nóbrega M.D.P.S. (2020). Mental illness in the general population and health professionals during COVID-19: A scoping review. Texto Contexto Enferm..

[3] Instituto de Salud Carlos III Situación de COVID-19 en España n° 54. https://www.isciii.es/QueHacemos/Servicios/VigilanciaSaludPublicaRENAVE/EnfermedadesTransmisibles/Documents/INFORMES/Informes%20COVID-19/Informe%20COVID-19.%20N%C2%BA%2054_25%20de%20noviembre%20de%202020.pdf.

[4] Huarcaya-Victoria J. (2020). Consideraciones sobre la salud mental en la pandemia de COVID-19. Rev. Peru. Med. Exp. Salud Pública.

[5] Ramírez-Ortiz J., Castro-Quintero D., Lerma-Córdoba C., Yela-Ceballos F., Escobar-Córdoba F. (2020). Consequences of the COVID-19 pandemic in mental health associated with social isolation. SciELO Prepr..

[6] Paiano M., Jaques A.E., Nacamura P.A.B., Salci M.A., Radovanovic C.A.T., Carreira L. (2020). Mental health of healthcare professionals in China during the new coronavirus pandemic: An integrative review. Rev. Bras. Enferm..

[7] Alvarez A.K.G., Almaguer A.Y.C., Santos E.D.Z. (2020). Management of health personnel’ psychological safety, in emergency situations by COVID-19 in the hospitable or isolation context. Rev. Cuba Enferm..

[8] Lai J., Ma S., Wang Y., Cai Z., Hu J., Wei N., Wu J., Du H., Chen T., Li R. (2020). Factors Associated with Mental Health Outcomes Among Health Care Workers Exposed to Coronavirus Disease 2019. JAMA Netw. Open.

[9] Chew N.W.S., Lee G.K.H., Tan B.Y.Q., Jing M., Goh Y., Ngiam N.J.H., Yeo L.L.L., Ahmad A., Khan F.A., Shanmugan G.N. (2020). A multinational, multicentre study on the psychological outcomes and associated physical symptoms amongst healthcare workers during COVID-19 outbreak. Brain Behav. Immun..

[10] Reger M.A., Piccirillo M.L., Buchman-Schmitt J.M. (2020). COVID-19, Mental Health, and Suicide Risk Among Health Care Workers: Looking Beyond the Crisis. J. Clin. Psychiatry.

[11] Torales J., Higgins M.O., Rios-gonzález C.M., Barrios I., Higgins M.O., Rios-gonzález C.M. (2020). Considerations on the mental health impact of the novel coronavirus outbreak (COVID-19). SciELO.

[12] Ornell F., Halpern S.C., Paim Kessler F.H., de Magalhães Narvaez J.C. (2020). The impact of the COVID-19 pandemic on the mental health of healthcare professionals. Cad. Saúde Pública.

[13] Rajkumar R.P. (2020). COVID-19 and mental health: A review of the existing literature. Asian J. Psyquiatr..

[14] Li Z., Ge J., Yang M., Feng J., Qiao M., Jiang R., Bi J., Chan G., Xu X., Wang L. (2020). Vicarious traumatization in the general public, members, and non-members of medical teams aiding in COVID-19 control. Brain. Behav. Immun..

[15] Vieta E., Pérez V., Arango C. (2020). Psychiatry in the aftermath of COVID-19. Rev. Psiquiatr. Salud. Ment..

[16] Wand A.P.F., Zhong B.L., Chiu H.F.K., Draper B., De Leo D. (2020). COVID-19: The implications for suicide in older adults. Int. Psychogeriatr..

[17] Huang Y., Zhao N. (2020). Generalized anxiety disorder, depressive symptoms and sleep quality during COVID-19 outbreak in China: A web-based cross-sectional survey. J. Psychiatr. Res..

[18] Shultz J.M., Cooper J.L., Baingana F., Oquendo M.A., Espinel Z., Althouse B.M., Marcelin L.H., Towers S., Espinola M., McCoy C.B. (2016). The Role of Fear-Related Behaviors in the 2013–2016 West Africa Ebola Virus Disease Outbreak. Curr. Psychiatry Rep..

[19] Liu C.Y., Yang Y.Z., Zhang X.M., Xu X., Dou Q.L., Zhang W.W., Cheng A.S.K. (2020). The prevalence and influencing factors in anxiety in medical workers fighting COVID-19 in China: A cross-sectional survey. Epidemiol. Infect..

[20] Centro de Investigación Biomédica de La Rioja Comité de Ética de la Investigación con Medicamentos de La Rioja (CEImLAR). https://www.cibir.es/es/plataformas-tecnologicas-y-servicios/ceimlar.

[21] Alexandre N.M.C., Gallasch C.H., Lima M.H.M., Rodrigues R.C.M. (2013). Reliability in the development and evaluation of measurement instruments in the health field. Rev. Eletr. Enferm..

[22] Coluci M.Z.O., Alexandre N.M.C., Milani D. (2015). Construção de instrumentos de medida na área da saúde. Ciênc. Saúde. Colet..

[23] Cunha C.M., de Almeida Neto O.P., Stackfleth R. (2016). Main psychometric evaluation methods of measuring instruments reliability. Rev. Aten. Saúde.

[24] Chen Q., Liang M., Li Y., Guo J., Fei D., Wang L., He L., Sheng C., Cai Y., Li X. (2020). Mental healthcare for medical staff in china during the COVID-19 outbreak. Lancet Psychiatry.

[25] Huang J.Z., Han M.F., Luo T.D., Ren A.K., Zhou X.P. (2020). Mental health survey of medical staff in a tertiary infectious disease hospital for COVID-19. Zhonghua Lao Dong Wei Sheng Zhi Ye Bing Za Zhi.

[26] Wang C., Pan R., Wan X., Tan Y., Xu L., Ho C., Ho R. (2020). Immediate psychological responses and associated factors during the initial stage of the 2019 coronavirus disease (COVID-19) epidemic among the general population in China. Int. J. Environ. Res. Public Health.

[27] Santamaría M.D., Etxebarria N.O., Rodríguez I.R., Alboniga-Mayor J.J., Gorrotxategi M.P. (2020). Impacto psicológico de la COVID-19 en una muestra de profesionales sanitarios españoles. Rev. Psiquiatr. Salud Ment..

[28] Wong T.W., Yau J.K., Chan C.L., Kwong R.S., Ho S.M., Lau C.C., Lau F.L., Lit C.H. (2005). The psychological impact of severe acute respiratory syndrome outbreak on healthcare workers in emergency departments and how they cope. Eur. J. Emerg. Med..

[29] Styra R., Hawryluck L., Robinson S., Kasapinovic S., Fones C., Gold W.L. (2008). Impact on healthcare workers employed in high-risk areas during the Toronto SARS outbreak. J. Psychosom Res..

[30] Lee S.M., Kang W.S., Cho A., Kim T., Park J.K. (2018). Psychological impact of the 2015 MERS outbreak on hospital workers and quarantined hemodialysis patients. Compr. Psychiatry.

